# Ezetimibe/Simvastatin 10/20 mg versus Rosuvastatin 10 mg in high-risk hypercholesterolemic patients stratified by prior statin treatment potency

**DOI:** 10.1186/1476-511X-9-127

**Published:** 2010-11-04

**Authors:** Margus Viigimaa, Helena Vaverkova, Michel Farnier, Maurizio Averna, Luc Missault, Mary E Hanson, Qian Dong, Arvind Shah, Philippe Brudi

**Affiliations:** 1 Tallinn University of Technology, Technomedicum, Ehitajate St. 5, 19086 Tallinn, Estonia; 2 3rd Department of Internal Medicine, Medical Faculty and University Hospital Olomouc, Olomouc, Czech Republic; 3 Point Medical - Rond Point de la Nation, Dijon, France; 4 Dipartimento di Medicina Clinica e Patologie Emergenti Policlinico Paolo Giaccone Università di Palermo, Palermo, Italy; 5 Department of Cardiology, St Jan Hospital Department of Cardiology, Bruges, Belgium; 6 Global Scientific and Medical Publications, Merck, 351 N Sumneytown Pike, North Wales, Pennsylvania 19454 USA; 7 Late Development Statistics, Merck, PO BOX 2000, Rahway, NJ 07065 USA; 8 Medical Affairs, Merck, P.O. BOX 100, Whitehouse Station, NJ 08889 USA

## Abstract

**Objective:**

This *post-hoc *analysis compared the lipid-altering efficacy of Ezetimibe/Simvastatin 10/20 mg (EZ/Simva) versus Rosuvastatin 10 mg (Rosuva) in patients stratified by statin potency/dose prior to randomization.

**Methods:**

Patients with elevated low-density lipoprotein cholesterol (LDL-C) despite prior statin treatment (n = 618) were randomized 1:1 to EZ/Simva 10/20 mg or Rosuva 10 mg for 6 weeks. Percent change from baseline in lipids and attainment of lipid targets were assessed within each subgroup (low potency n = 369, high potency n = 249). Consistency of the treatment effect across subgroups was evaluated by testing for treatment-by-subgroup interaction. No multiplicity adjustments were made.

**Results:**

Significant treatment-by-subgroup interaction occurred for LDL-C (p = 0.013), total cholesterol (p = 0.025), non-HDL-C (p = 0.032), and apolipoprotein B (p = 0.016) with greater between-treatment differences in favor of EZ/Simva observed in patients from the high potency stratum vs low potency stratum. Individual and triple target attainment was higher for Eze/Simva compared with Rosuva in both strata.

**Conclusions:**

Compared with Rosuva, switching to EZ/Simva provided greater reductions in LDL-C, total cholesterol, non-HDL-C and apolipoprotein B and higher target attainment in patients on prior statin treatment, regardless of potency, although patients treated with higher potency statins prior to randomization experienced greater between treatment differences in favor of EZ/Simva.

**Trial Registration:**

Registered at ClinicalTrials.gov: NCT00479713

## Background

Low-density lipoprotein cholesterol (LDL-C) is the primary target of therapy in patients with hypercholesterolemia[1,2]. If therapeutic lifestyle changes do not lower LDL-C to recommended levels, interventions such as HMG-CoA reductase inhibitors (statins) are indicated for further LDL-C reductions. Rosuvastatin has demonstrated more effective LDL-C reductions across its dose range than other statins across their dose ranges and greater lipid-lowering effects compared with the other marketed statins[3-5]. However, despite the substantial lipid-lowering associated with the use of statins, LDL-C reductions beyond that achieved by currently used statin therapies--even those considered to be of highest potency--are recommended for a considerable number of patients[6-9]. Many patients at high cardiovascular risk and/or with severely elevated LDL-C do not achieve the rigorous treatment targets recommended by European, Canadian and US guidelines using established statin treatment regimens[1,2,10,11].

Clinical trial results have demonstrated that combining ezetimibe with a statin more effectively lowers LDL-C than therapy with either of the individual components alone[9,12]. In a study of ezetimibe combined with simvastatin vs rosuvastatin in treatment-naïve patients, at each dose comparison and across doses, ezetimibe/simvastatin reduced LDL-C levels significantly more than rosuvastatin[13]. That trial utilized a placebo run-in, which may not reflect usual clinical practice in which high cardiovascular risk patients are usually already being treated with a statin and require dose adjustments or treatment changes to achieve desired lipid levels. In the trial presented here patients were treated prior to randomization with statins of varying potency but had not achieved LDL-C <100 mg/dL[14]. The results of the primary analysis of this trial demonstrated significantly greater reductions in LDL-C and other lipid levels and significantly greater achievement of prespecified LDL-C treatment targets with ezetimibe/simvastatin 10/20 mg (EZ/Simva) versus rosuvastatin 10 mg (Rosuva) in the overall population following 6 weeks of treatment[14]. The objective of this post hoc analysis was to compare the lipid-altering efficacy of two treatment regimens recommended for clinical use. Analyses were done in patients grouped by statin potency/dose prior to randomization.

## Methods

The methods for this study were previously published[14]. Briefly, this was a multicenter, randomized, double-blind, parallel-group study conducted at 85 sites in 10 countries in Europe between March 2007 and March 2008. The study protocol was approved by the appropriate ethics committees and institutional review boards at each site, and all patients provided written informed consent before any study procedures were administered.

### Patients

Men and women 18 to 80 years of age with documented hypercholesterolemia [LDL-C ≥100 and ≤190 mg/dL at screening and ≥100 and ≤160 mg/dL at randomization] and high cardiovascular risk who were taking a stable daily dose of atorvastatin 10 or 20 mg; fluvastatin 80 mg; pravastatin 40 mg; rosuvastatin 5 mg; or simva 20 or 40 mg for at least 6 weeks prior to the study randomization were enrolled in the study. Patients were considered high cardiovascular risk if they had a history of CHD (i.e. stable and unstable angina, revascularization procedure, myocardial infarction, documented silent myocardial ischemia), or with established vascular atherosclerotic disease (i.e. peripheral vascular disease, ischemic stroke); type 2 diabetes without a history of vascular disease and with high cardiovascular risk (i.e. renal impairment [proteinuria >300 mg/24 h or creatinine clearance (standardized for body surface area) <1.002 ml/s] and/or at least two CHD risk factors per Framingham risk calculation); or CHD risk >20% over 10 years as determined by the Framingham risk calculation. All patients must have had fasting triglyceride levels <350 mg/dL, alanine aminotransferase (ALT) and aspartate aminotransferase (AST) levels ≤1.5 × the upper limit of normal (ULN), and creatine kinase (CK) levels ≤ 3 × ULN at baseline.

Patients were excluded if they had conditions or medications other than reported statin medications that could have affected lipid levels; active liver disease; congestive heart failure; poorly controlled diabetes [hemoglobin A1c (HbA1c) ≥8.5%]; uncontrolled hypertension (systolic >160 mmHg or diastolic >100 mmHg); or impaired renal function (creatinine ≥176.8 mmol/L). Patients taking prescription and/or over-the counter-drugs with the potential for significant lipid effects (other than study drug or above listed statins), or with potential drug interactions with the statins were also excluded from the study.

### Treatments

At screening, patients were enrolled in the open-label run-in phase during which they continued to receive their current dose/brand of statin medication for 6 weeks. At randomization, eligible patients received either EZ/Simva 10/20 mg/day or rosuvastatin 10 mg/day for 6 weeks. Patients were counseled regarding diet and exercise at each clinic visit.

### Efficacy measures

In patients grouped by potency of statin at baseline (low potency stratum included simvastatin 20 mg, pravastatin 40 mg, fluvastatin 80 mg, atorvastatin 10 mg and high potency stratum included simvastatin 40 mg, atorvastatin 20 mg, rosuvastatin 5 mg), the percent change from baseline in LDL-C, total cholesterol, HDL-C, non-HDL-C, triglycerides, hs-CRP, LDL-C/HDL-C ratio, total cholesterol/HDL-C ratio and Apo B were assessed. In addition, the percentage of patients achieving LDL-C <100 mg/dL, <77 mg/dL, or <70 mg/dL; non-HDL-C <130 mg/dL or <100 mg/dL; Apo B <90 or <80 mg/dL; and achievement of the combination of LDL-C <100 mg/dL, non-HDL-C <130 mg/dL *and *Apo B <90 mg/dL was assessed.

### Statistical Methods

The analyses were based on the Full-Analysis-Set (FAS) population which included all patients who received randomized treatment and had a baseline and at least one measurement performed after treatment start. The efficacy analyses for LDL-C, total cholesterol, HDL-C, non-HDL-C, LDL-C/HDL-C ratio, total cholesterol/HDL-C ratio and Apo B were analyzed using a parametric ANOVA model. The consistency of the treatment effect across the two strata subgroups (low/high potency) was assessed through the significance of the treatment-by-subgroup interaction term in the ANOVA model with terms for treatment, baseline efficacy variable (categorized based on quartiles), study center, stratum subgroup and the treatment-by-stratum subgroup interaction. The least-square (LS) means and 95% confidence intervals (CIs) within each subgroup level were used to quantify the difference between treatment groups from the above ANOVA model (except for the last two terms involving subgroup). Consistency of the treatment effects on triglycerides and hs-CRP were assessed through the significance of the treatment-by-subgroup interaction term in the ANOVA model on ranks of these efficacy variables with terms for treatment, baseline triglycerides (or hs-CRP) categorized based on quartiles, study center, stratum potency status and the treatment-by-stratum subgroup interaction. The difference between treatment groups was quantified using the difference in medians and 95% CIs using Hodges-Lehmann estimates within each subgroup.

The percentages of patients that achieved specified targets at endpoint were analyzed via a logistic regression model. The consistency of the treatment effect across the two strata subgroups was assessed through the significance of the treatment-by-subgroup interaction term in the logistic regression model with terms for treatment, baseline lipid variable as continuous variable, stratum status, and treatment-by-stratum subgroup interaction. Odds ratio estimates and 95% CIs were derived from the logistic regression model with similar terms except for the last two involving subgroups. Due to the exploratory nature of this analysis, no multiplicity adjustments nor power computation were employed.

## Results

### Baseline

The flow of patients through the study was previously published[14]. In the EZ/Simva group (N = 314) there were 189 patients taking low-potency statins and 125 patients taking high-potency statins at baseline. In the rosuvastatin group (N = 304), there were 180 patients taking low-potency statins and 124 patients taking high-potency statins at baseline. Within each potency strata group, baseline demographic and clinical characteristics were generally well-balanced, although there were more patients with diabetes in the low-potency statin group compared with the high-potency statin group (Table 1 and Table 2).

**Table 1:** Baseline demographics and risk factors

	Low Potency*			**High Potency**^†^		
	**E/S 10/20** **(n = 189)**	**R 10** **(n = 180)**	**Total** **(N = 369)**	**E/S 10/20** **(n = 125)**	**R 10** **(n = 124)**	**Total** **(N = 249)**

Male, n (%)	74 (39.2)	77 (42.8)	151 (40.9)	55 (44.0)	42 (33.9)	97 (39.0)
Female, n (%)	115 (60.8)	103 (57.2)	218 (59.1)	70 (56.0)	82 (66.1)	152 (61.0)
Mean Age, y (SD)	63.4 (9.3)	63.5 (10.6)	63.5 (10.0)	62.9 (10.5)	62.4 (9.1)	62.7 (9.8)
Race n (%)						
White	189 (100)	180 (100)	369 (100)	125 (100)	122 (98.4)	247 (99.2)
Other					2 (1.6)	2 (0.8)
BMI <30 kg/m^2‡ ^(%)	128 (67.7)	134 (74.4)	262 (71.0)	88 (70.4)	87 (70.2)	175 (70.3)
BMI ≥30 kg/m^2‡ ^(%)	61 (32.3)	46 (25.6)	107 (29.0)	36 (28.8)	37 (29.8)	73 (29.3)
**Risk factors **[n (%)]						
CHD	90 (47.6)	84 (46.7)	174 (47.2)	62 (49.6)	60 (48.4)	122 (49.0)
Diabetes	60 (31.7)	52 (28.9)	112 (30.4)	35 (28.0)	26 (21.0)	61 (24.5)
Ex-smoker	37 (19.6)	44 (24.4)	81 (22.0)	40 (32.0)	26 (21.0)	66 (26.5)
Non smoker	94 (49.7)	87 (48.3)	181 (49.1)	50 (40.0)	51 (41.1)	101 (40.6)
Smoker	58 (30.7)	49 (27.2)	107 (29.0)	35 (28.0)	47 (37.9)	82 (32.9)

E = ezetimibe; BMI = body mass index; CHD = coronary heart disease; n = number; R = rosuvastatin; S = simvastatin; SD = standard deviation; y = year

*Low potency stratum included: simvastatin 20 mg, pravastatin 40 mg, fluvastatin 80 mg, atorvastatin 10 mg

^†^High potency stratum included: simvastatin 40 mg, atorvastatin 20 mg, rosuvastatin 5 mg

^‡^Missing data for 1 patient in the high potency EZ/Simva group

**Table 2:** Baseline clinical characteristics

	Low Potency			High Potency		
**Mean (SD)**	**E/S 10/20** **(n = 189)**	**R 10** **(n = 180)**	**Total** **(N = 369)**	**E/S 10/20** **(n = 125)**	**R 10** **(n = 124)**	**Total** **(N = 249)**

LDL-C (mg/dL)	125.2 (16.3)	124.2 (16.7)	124.7 (16.5)	121.2 (15.7)	126.7 (16.6)	123.9 (16.3)
HDL-C (mg/dL)	55.9 (15.1)	55.0 (13.7)	55.4 (14.4)	54.7 (12.9)	55.0 (13.6)	54.9 (13.2)
non-HDL-C (mg/dL)	153.0 (21.4)	150.5 (21.1)	151.8 (21.2)	150.4 (21.0)	156.0 (21.6)	153.1 (21.5)
TC (mg/dL)	208.9 (22.7)	205.5 (22.6)	207.2 (22.7)	205.1 (21.2)	210.9 (23.4)	208.0 (22.4)
TG (mg/dL)*	125.0 (72.6)	116.0 (68.8)	121.0 (70.7)	134.5 (75.3)	135.0 (77.7)	135.0 (75.3)
Apo B (mg/L)	119.4 (19.5)	115.4 (21.7)	117.4 (20.7)	119.3 (20.1)	122.4 (18.5)	120.8 (19.4)
hs-CRP (mg/L)*	1.7 (2.7)	1.4 (2.2)	1.6 (2.5)	1.4 (2.1)	1.6 (2.9)	1.6 (2.4)
LDL-C/HDL-C	2.4 (0.7)	2.4 (0.7)	2.4 (0.7)	2.3 (0.6)	2.4 (0.7)	2.4 (0.6)
TC/HDL-C	3.9 (0.9)	3.9 (0.9)	3.9 (0.9)	3.9 (0.9)	4.0 (0.9)	4.0 (0.9)

Apo B = apolipoprotein B; E = ezetimibe; hs-CRP = high-sensitivity C-reactive protein; LDL-C = low-density lipoprotein cholesterol; HDL-C = high-density lipoprotein cholesterol; R = rosuvastatin; S = simvastatin; TG = triglycerides; TC = total cholesterol

*presented as median values with standard deviation (SD) calculated by (Q3-Q1)/1.075

### Efficacy endpoints

**Figure 1:**
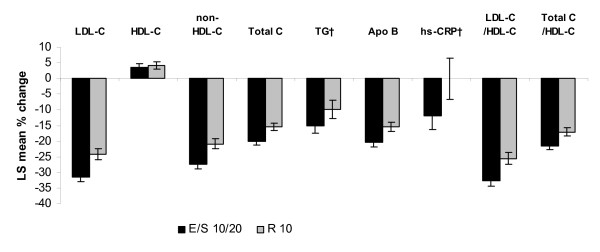
Percent change in lipid and hs-CRP levels after 6 weeks of treatment in patients treated with low-potency statins* at baseline. *Low potency stratum included: simvastatin 20 mg, pravastatin 40 mg, fluvastatin 80 mg, atorvastatin 10 mg. ^†^Presented as median values. Apo B = apolipoprotein B; E = ezetimibe; hs-CRP = high-sensitivity C-reactive protein; LDL-C = low-density lipoprotein cholesterol; HDL-C = high-density lipoprotein cholesterol; R = rosuvastatin; S = simvastatin; TG = triglycerides; Total C = total cholesterol

**Figure 2:**
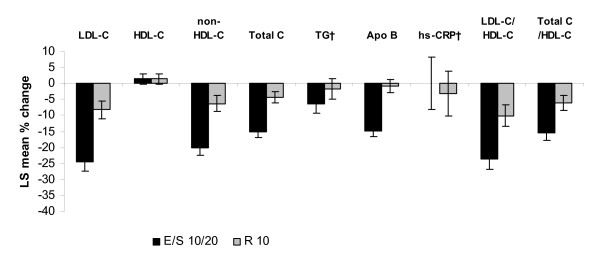
Percent change in lipid and hs-CRP levels after 6 weeks of treatment in patients treated with high-potency statins* at baseline. *High potency stratum included: simvastatin 40 mg, atorvastatin 20 mg, rosuvastatin 5 mg ^†^Presented as median values Apo B = apolipoprotein B; E = ezetimibe; LDL-C = low-density lipoprotein cholesterol; HDL-C = high-density lipoprotein cholesterol; hs-CRP = high sensitivity C-reactive protein; R = rosuvastatin; S = simvastatin; TG = triglycerides; Total C = total cholesterol

Both treatment regimens resulted in improvements in LDL-C, non-HDL-C, total cholesterol, triglyceride, Apo B, LDL-C/HDL-C ratio and total cholesterol/HDL-C ratio levels after 6 weeks of treatment, although the percent change from baseline was greater in patients treated with EZ/Simva 10/20 mg for all endpoints compared with patients treated with rosuvastatin 10 mg in both potency strata groups (Figure 1 and Figure 2). Significant treatment-by-stratum interactions were observed for LDL-C (p = 0.013), total cholesterol (p = 0.025), non-HDL-C (p = 0.032), and Apo B (p = 0.016), with greater between treatment differences in favor of EZ/Simva observed in patients from the high-potency stratum compared with the low-potency stratum (Figure 1 and Figure 2). Treatment effects were consistent across the two subgroups (low-potency/high-potency) with the overall population in HDL-C, LDL-C/HDL-C, total cholesterol/HDL-C ratio, hs-CRP and triglyceride levels.

**Figure 3:**
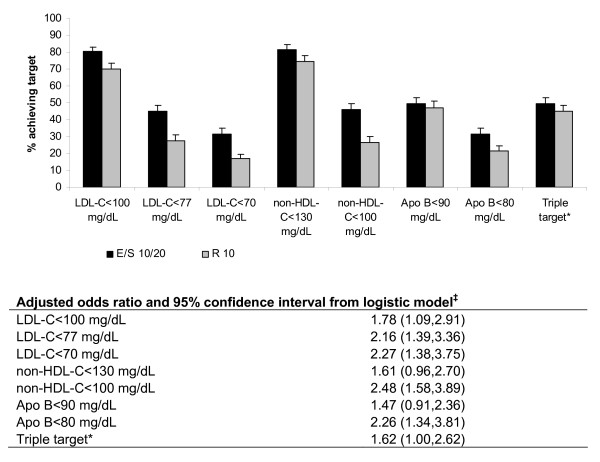
**Percent of patients achieving lipid targets at 6 weeks (treated with low-potency statins**^**† **^**at baseline)**. *Triple target = LDL-C <100 mg/dL and non-HDL-C <130 mg/dL and Apo B <90 mg/dL ^†^Low potency stratum included: simvastatin 20 mg, pravastatin 40 mg, fluvastatin 80 mg, atorvastatin 10 mg^‡^Ratio of the predictive odds of attaining target on EZ+Simva versus Rosuvastatin based on the logistic model (fitted within each subgroup) with terms for treatment and baseline values of the variable being modeled. Apo B = apolipoprotein B; E = ezetimibe; LDL-C = low-density lipoprotein cholesterol; non-HDL-C = non-high-density lipoprotein cholesterol; R = rosuvastatin; S = simvastatin

**Figure 4:**
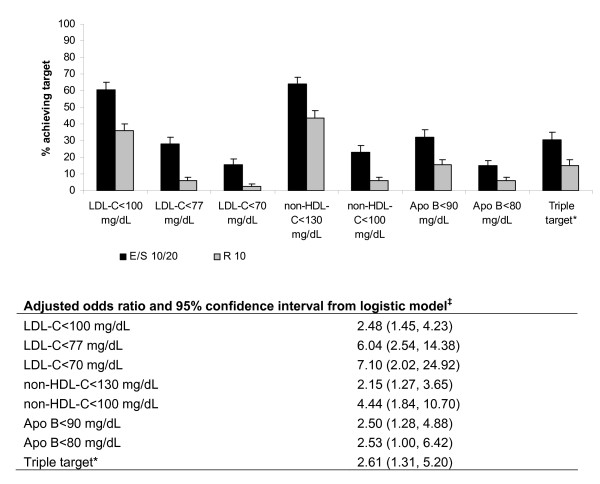
**Percent of patients achieving lipid targets at 6 weeks (treated with high-potency statins**^**† **^**at baseline)**. *Triple target = LDL-C <100 mg/dL and non-HDL-C <130 mg/dL and Apo B <90 mg/dL ^†^High potency stratum included: simvastatin 40 mg, atorvastatin 20 mg, rosuvastatin 5 mg ^‡^Ratio of the predictive odds of attaining target on EZ+Simva versus Rosuvastatin based on the logistic model (fitted within each subgroup) with terms for treatment and baseline values of the variable being modeled. Apo B = apolipoprotein B; E = ezetimibe; LDL-C = low-density lipoprotein cholesterol; non-HDL-C = non-high-density lipoprotein cholesterol; R = rosuvastatin; S = simvastatin

Higher percentages of patients achieved the specified treatment targets in the EZ/Simva group compared with patients treated with rosuvastatin in both potency groups (Figures 3 and Figure 4). The magnitude of between-group differences was generally greater in the high-potency statin group compared with the low-potency statin group. In the low-potency group, the predictive odds of attaining all individual specified lipid levels and the triple combination were greater in patients treated with EZ/Simva compared with rosuvastatin (see table under Figure 3). In the high-potency group, the predictive odds of attaining all individual specified lipid levels and the triple combination were greater in patients treated with EZ/Simva compared with rosuvastatin (see table under Figure 4). In the high-potency group, the predictive odds of attaining the lipid levels were higher compared with those in the low-potency groups, although the treatment effects on attainment of specified levels for LDL-C, non-HDL-C, Apo B and the triple combination target were generally consistent across the two potency groups.

## Discussion

This post hoc analysis demonstrated that in patients with hypercholesterolemia at high risk for CHD who had not attained specified LDL-C treatment levels, switching to EZ/Simva 10/20 from high- or low-potency statins resulted in greater further reductions in most lipid levels compared with switching to rosuvastatin 10 mg for 6 weeks. In addition, switching to EZ/Simva 10/20 from high- or low-potency statins results in superior achievement of specified lipid targets vs rosuvastatin 10 mg treatment.

The mean duration of hypercholesterolemia in patients enrolled in this study was 8 years, which suggests that patients were either candidates for or had received long-term treatment prior to study start but had not yet achieved suggested therapeutic targets. One would expect that with high-potency statin treatment, over time patients would achieve lower lipid levels than with low-potency statin treatment. However, in this population of patients who had been treated with statins for a minimum of 6 weeks prior to study start, and quite possibly for much longer than 6 weeks, baseline lipid levels were similar between the two statin potency groups. Therefore, even with what may be considered more intensive statin monotherapy, a considerable number of patients did not achieve therapeutic targets on statin monotherapy.

Of note the magnitude of response to the combination treatment appeared to be greater in terms of percent change from baseline in patients in the high-potency group vs patients in the low-potency group. Moreover, the odds of attaining most specified lipid levels (especially the LDL-C targets) in the high-potency group was double or triple the odds of attaining specified lipid levels in the low-potency group. These results suggest that some subjects in the high-potency group may be poor responders to statins. One reason for poor response to statin therapy and inadequate goal achievement may be inter-individual variability in LDL-C lowering, which has been reported with high-potency statins[15]. Similar inter-individual variability has also been shown with ezetimibe[16], but to date, even if no studies have been published demonstrating this same variability with the combination treatment, the complimentary mechanisms targeting the synthesis and absorption of cholesterol may explain the better efficacy in patients who are poor responders to statins.

Although the mechanisms are poorly understood, research has begun to illuminate potential reasons why the response to statins and other drugs varies widely among individuals. Non-modifiable factors that may impact statin response include sex and age but these associations have not been fully elucidated. Variability associated with age may be attributed to poor compliance[17] or altered kidney function, which may be remedied through close monitoring and dose adjustment[18]; and hormone fluctuations may play a role in the variability observed in the elderly and between sexes[19]. Some differences in statin response may be attributed to genetic variations in pharmacokinetic- and pharmacodynamic-related genes. Genetic variations in LDL receptor expression were shown to be associated with diminished statin response (i.e., diminished effects of statins on *in vivo *lipid reductions and on *in vitro *LDL receptor induction); and similarly, attenuated statin-mediated changes were observed in subjects with genetic mutations in HMG CoA reductase[20].

Without solid scientific evidence and pharmacogenetic tests to inform clinical decisions, individual patient response to statin monotherapy is likely the only way to assess the need to modify dosages or therapies to attain lipid goals. Although statins are typically considered the first line of therapy for lipid-lowering, this can prove to be a time-consuming and expensive endeavor for patients who respond suboptimally to statins. These patients are candidates for combination therapy with complimentary mechanisms of action, such as ezetimibe added to statin; and clinical trial results support an approach to lipid-lowering that targets the synthesis and the absorption of cholesterol in patients with suboptimal response to statin monotherapy [14,21,22]. Using a Markov model, the use of ezetimibe in combination with simvastatin was projected to be a cost-effective alternative to continuing or doubling the dose of a statin,[23] and the results of the present analyses further support the use of EZ/Simva 10/20 mg vs rosuvastatin 10 mg, a high potency statin, for improving hypercholesterolemia in patients who had not achieved LDL-C targets on previous statin monotherapy, regardless of potency. Studies to demonstrate the efficacy of ezetimibe/statins vs statins on clinical outcomes are under way[24].

## Competing interests

MV: none. HV: Speakers' bureau and consultant and/or advisory board member: Pfizer, AstraZeneca, Solvay, Merck, Teva, Zentiva. MF: Research grant support, honoraria and consultant and/or advisory board membership: AstraZeneca, Genzyme, Kowa, Merck, Novartis, Pfizer, sanofi-aventis, SMB, Solvay, Takeda. MA: none. LM: Speakers' bureau and consultant and/or advisory board member: Merck, Boehringer-Ingelheim, AstraZeneca. QD, AS, MEH, PB: Employees of Merck and may own stock or hold stock options in the company.

## Authors' contributions

MV, HV, MF, MA, and LM, collected or assembled the data, provided study materials or patients, interpreted the results, wrote sections of the initial draft, and provided substantive suggestions for revision on subsequent iterations of the manuscript. PB conceived, designed or planned the study, collected or assembled the data, interpreted the results, and provided substantive suggestions for revision on subsequent iterations of the manuscript. MEH interpreted the results, wrote sections of the initial draft, provided substantive suggestions for revision on subsequent iterations of the manuscript, and provided administrative and logistical support. QD and AS conceived, designed or planned the study, performed or supervised the analyses, interpreted the results, wrote sections of the initial draft, provided substantive suggestions for revision on subsequent iterations of the manuscript, and provided statistical expertise. All authors reviewed and approved the final version of the paper.
